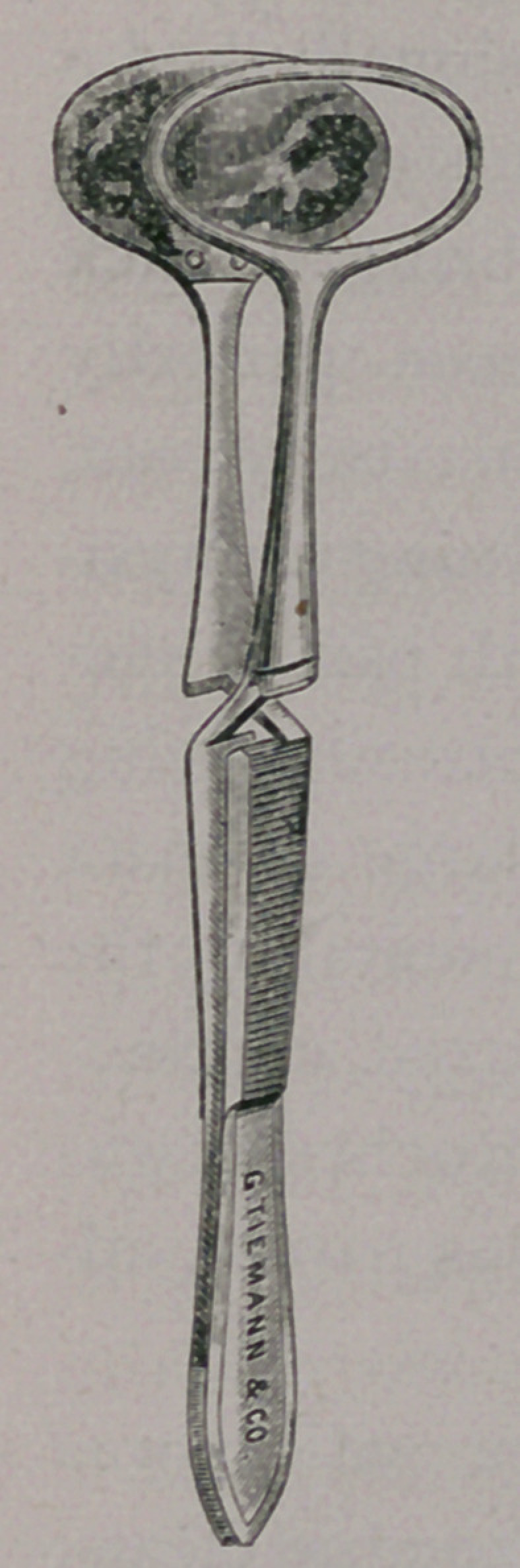# Self-Adjusting, Shell-Plate Entropium Forceps

**Published:** 1882-04

**Authors:** 

**Affiliations:** Cornell University, Ithaca, N. Y.


### Self-Adjusting, Shell-Plate Entropium Forceps.

By Dr. Rœhrig, Cornell University, Ithaca, N. Y.

This instrument was originally designed to be an improvement on Desmarres' forceps. Its chief excellence consists:

1. In the substitution of a soft and yielding *shell plate* for the hard and rigid steel. This shell plate, which has thereafter been generally adopted in the construction of other instruments of a similar use and nature, I was at that time the first to introduce as an improvement in surgery.

2. I thought best to do away altogether with the screw (of Desmarres' instrument), and substituted in its place two crossing branches with a spring of gentle, moderate power.

It may indeed be said in behalf of the screw that only by means of the same can the degree of pressure be regulated and made more or less intense; still, such a difference in degree is here hardly requisite, an average compression and a steady hold or fixation being all that is really needed, and this is obtained by the contrivance just mentioned. It secures a moderate degree of pressure and steadiness, and instead of directing, during the operation, any further attention to the screw —which now and then necessitates some loss of time, inconvenience and trouble—the instrument has become, as it were, in a measure *self-adjusting*.

The advantages here set forth seem to have been fully appreciated by the profession, since the instrument became in a short time after its introduction quite a favorite with ophthalmic surgeons, and has been in constant demand. Its usefulness and convenience having thus been tested and acknowledged, it seems well to make the above statement, as I had heretofore neglected to give the matter any due publicity.

## Figures and Tables

**Figure f1:**